# Using Lung Organoids to Investigate Epithelial Barrier Complexity and IL-17 Signaling During Respiratory Infection

**DOI:** 10.3389/fimmu.2019.00323

**Published:** 2019-02-28

**Authors:** Giuseppe Paolicelli, Antonella De Luca, Shyam S. Jose, Martina Antonini, Irene Teloni, Jan Fric, Teresa Zelante

**Affiliations:** ^1^Department of Experimental Medicine, University of Perugia, Perugia, Italy; ^2^Center for Translational Medicine, International Clinical Research Centre, St. Anne's University Hospital Brno, Brno, Czechia

**Keywords:** IL-17 immunity, epithelial barrier, lung infection, *Aspergillus fumigatus*, lung organoids

## Microbial Causes of Respiratory Tract Infections

The respiratory system is the first point of contact with airborne microbial compounds. Consequently, lung mucosal immunity has been extensively studied to understand the mechanisms of host resistance to respiratory infections. The lungs exhibit highly active innate and adaptive mucosal immune mechanisms: they are infiltrated with a wide spectrum of immune cells in steady state and possess the capacity to recruit vast numbers of infiltrating cells upon infection or encounter with inflammatory stimuli. Despite the existence of such protective mechanisms, respiratory tract infections (RTIs) with epidemic and pandemic potential are one of the most common causes of morbidity and mortality worldwide. In recent years, studies using new lung culture systems, such as air liquid interface (ALI), spheroids, tissue explants and advances in DNA sequencing technology have helped identify that the upper and lower respiratory tracts represent distinct biomes in terms of their commensal microorganism colonization, immune barriers and host defense mechanisms (1–3). Most lower respiratory tract infections (LRTIs) cause bronchitis, bronchiolitis and pneumonia as a result of *Streptococcus pneumonia* or *Haemophilus Influenzae* infection. In children, respiratory viruses are responsible for an enormous amount of serious LRTIs (4, 5). In addition, most upper respiratory tract infections are of viral etiology (6). Fungal infections of the lower respiratory tract are also typically caused by pathogenic dimorphic fungi (7). In addition, opportunistic fungi as *Aspergillus fumigatus* commonly cause pneumonia. There is an extraordinary need to better understand human respiratory tract infections, as LRTI represent one of the ten most common causes of death in the world (8).

Technical limitations are inherent with pneumonia animal models and *in vitro* lung infections modeled using immortalized cell lines. In particular, for *in vivo* models, lung anatomy, namely the distribution of the bronchial glands, differs between rodents and humans, and complex processes such as mucus production, or organization of the epithelial barrier are not accurately reproduced experimentally. For *in vitro* lung infections, it is not possible to reproduce *in vivo*-like architecture, the microenvironment, the pulmonary cell complexity in composition. Moreover, bronchial epithelial cells lack cilia and tight junctions. Although lung epithelial barrier cell signaling is today more deeply understood, it has still not been fully evaluated in reproducible lung infection models.

Recent advances in the stem-cell field, including the generation of protocols allowing tissue differentiation from induced pluripotent stem cells (iPSCs), have provided new opportunities to study host–pathogen interactions in a human experimental system that maintains controlled tissue complexity. For this reason, recently developed techniques now allow for innovative and more meaningful investigations of 3D human lung tissue. Here we outline the complexity of the epithelial barrier to opportunistic microbes and the new 2D and 3D lung models of infection, and explain how these models may be used to improve our knowledge on epithelial cell signaling events upon infection.

## Complexity of the Lung Epithelial Barrier

Epithelial cells represent the first point of contact for opportunistic microbes or pathogens in the respiratory tract (9). The lung mucosa senses infection through pattern recognition receptors expressed by the airway epithelia (10–12), alveolar cells (13, 14), and mesenchymal stem cells (15, 16). Several cell types then orchestrate mucosal barrier immunity: club cells, ciliated cells, basal cells, goblet cells and neuroendocrine cells as tuft cells decorate the proximal airways, while type-1 and type-2 alveolar cells populate the distal epithelium. The lungs can also be divided into a conducting zone and a respiratory zone, which are populated by different progenitor cell types. The conducting zone is abundant in basal cells (17, 18), airway secretory club cells and lineage-negative epithelial cells (18). The respiratory zone is mainly populated by alveolar type II cells (AEC II) that can proliferate and act as progenitor cells, replacing AECII and AECI cells (19).

The complex barrier functions executed by the lung epithelial layer, including mucociliary clearance and antimicrobial production, cooperate to clear inhaled pathogens. Unsurprisingly, gaining a clear understanding of the lung epithelial barrier has been restrained by this described complexity of the lung organization and its underlying cell types. Early research strategies based on immortalized airway epithelial cells or lung primary cells thus may not replicate the conditions where inhaled microorganisms become pathogens that trigger infections.

## Developing 2D and 3D Tools to Mimic Lung Structure

*In vivo* lung epithelial barrier experiments are challenging: dissecting the roles of individual cell types is complex due to the heterogeneity of the lung and the lack of specific cell markers. Much research has thus utilized the 2D ALI system and immortalized, lung cancer-derived cell culture approaches to study airway epithelial barrier–pathogen interactions *in vitro* (20, 21). The ALI system has been successfully used to differentiate progenitor cells, such as primary bronchial epithelial cells, into the corresponding airway tract upon exposure to the appropriate culture conditions (22). For example, basal cells cultured in an ALI system, can differentiate into a pseudo-stratified epithelium containing ciliated, goblet and basal cells (23). So far, this method has helped elucidate the transcriptomic profile of basal cells (24) and the impact of virus-infected basal cells on epithelium development (25). The limits of these “conventional” approaches, however, are namely the lack of tissue architecture; indeed, they are not able to faithfully recapitulate the phenotypic and morphological characteristics of the native epithelium (Table 1).

**Table 1:** Comparison between advanced cell culture techniques for lung infection studies.

**Tecnique**	**Advantage**	**Disadvantage**
Air Liquid Interface	Easy to use protocol	Not *in vivo*-like architecture
	High reproducibility	Lack of morphological characteristics of the native epithelium
	Compliant with high-troughtput screening (HTS)/ High Content Screening (HCS)	
	Patient specific	
	Low cost system	
Lung-on-a-Chip	Physiological environment (perfusion, stretch)	Not adaptable to HTS
	Alveolar–capillary interface	High cost
	Surfactant production and electrical resistance	Difficult to manufacture
Lung organoids	*in vivo*-like complexity and architecture	Lack of vascolature and immune cells
	Histological structures and function of native tissue	Require meticulous maintenance
	Scalable to different plate format	
	High reproducibility	
	Compliant with HTS/HCS	
	Patient specific	

To overcome such limitations of the ALI 2D system, an ALI 3D culture system has been developed, in which human bronchial epithelial cells are cultured on a permeable membrane submerged in media supplemented with stromal cells or growth factors (26).

A precursor to lung organoid culture was the development of a “Lung-on-a-Chip”: a microphysciological device that replicates on a chip the functional unit of the breathing lung. This system is based on communication between alveolar and endothelial cells through a microporous elastomeric membrane. The alveolar cells located in the upper chamber, are exposed to air and fed by endothelial cells grown in the bottom layer. Although this Lung-on-a-Chip system has been used for drug discovery and toxicology studies (27), it is not possible to recreate the lung architecture, which has a central role in various physiological functions (Table 1) (28).

**Figure 1:**
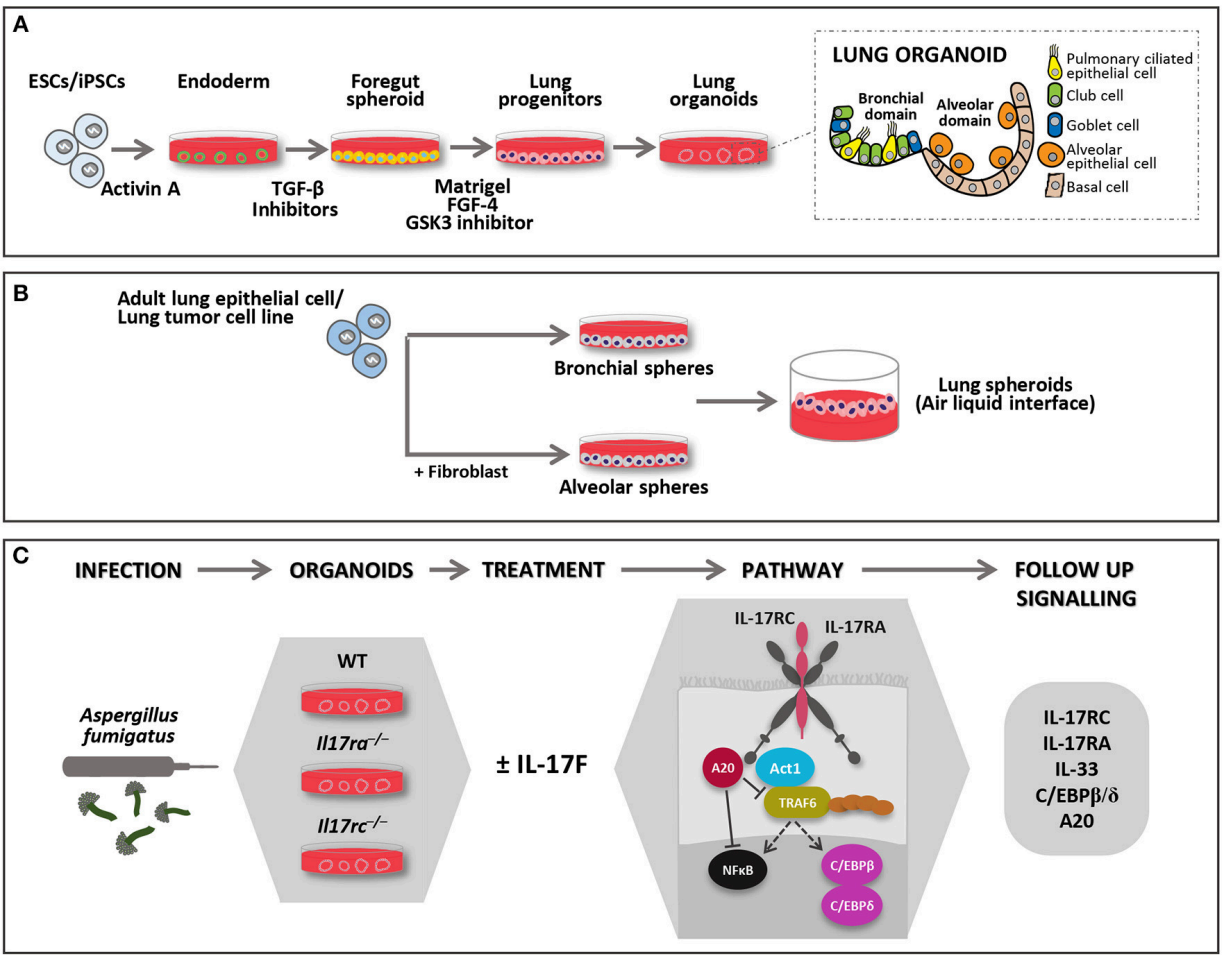
Models of 3D lung organoid infections. Lung organoids can be developed from iPSCs or adult stem cells (upper panel). **(A)** iPSCs derived lung organoids: cells are differentiated into endoderm by Activin A and further to anterior foregut followed by lung progenitor spheroids through the activation and inhibition of several signaling pathways. The progenitor spheroids are further embedded in matrigel to develop 3D lung organoids when supplemented with appropriate growth factors, which resemble lung tissue in morphology and function. They form the bronchial and alveolar like domains of the lungs and has both functional (epithelial cells) and supportive (basal mesenchymal cells) pulmonary tissue. **(B)** Adult lung progenitor cells can form spheroids, which can be further cultured on ALI to mimic lung environment. Spheroids do not form the exact morphology and lack some of the functional cell types. They can either form alveolar or bronchial branch based on various protocols, some of them require co-culture with support cells. **(C)** In the lower panel, the description of an experimental model of RTIs where lung organoids derived from different genotypes may be injected with Aspergillus fumigatus. The model may be used to study IL-17R signaling pathways in 3D system where the complex role of IL-17F may be studied. FGF-4 (Fibroblast growth factor 4); GSK3 (Glycogen synthase kinase 3); TGF-β (Transforming growth factor beta 1).

Over the past 15 years, new *in vitro* strategies have facilitated the production of miniature 3D structures, known as “mini organs” or organoids. Organoids are multi-cellular, stem-cell-derived systems in which cells spontaneously self-organize into properly differentiated, functional cell types that resemble *in vivo* counterparts and recapitulate the key features of the entire organ (Table 1). Organoids can help dissecting the role of individual cell types because are deprived of immune cells and endothelial cells. The overall approach is based on using hydrogels containing a gelatinous mixture, such as laminin and collagen, to mimic the extracellular matrix and self-organizing iPSCs. The human adult lung stem cells that are essential for epithelial renewal and tissue repair have proven capable of generating such 3D human lung organoids when cultured in the appropriate differentiating conditions. Adult stem cells, once isolated from the peripheral tissue, are usually cultured in enriched medium in the presence of a hydrogel scaffold that provides structural support and mediates instructive signaling for cell polarization, retention and mobilization. This organoid medium needs to be changed every 4 days, and the organoids must be passaged every 2 weeks. After 3 weeks of culture, starting from adult stem cells, the first small organoids reproduce the faithful microanatomy of the lung and recapitulate some specific lung functions (Figure 1) (29). The protocols using human tissue stem cells and iPSCs have been developed further to study organ-related pathologies and ontogeny (30–32). Over the past decade, although lung organoids have been used for much translational research, such as lung engraftment (33), the main application has been *in vitro* disease modeling.

## Using Organoids to Model RTIs

Access to the organoid lumen for experimental perturbation is challenging; thus, many researchers add bacteria to the supernatants of organoid-derived 2D cultures to monitor host–pathogen interactions. Cutting-edge technologies, however, now permit microinjection of microbes into the organoid lumen (34), allowing host–microbiota interplay within the 3D structure. More recently, a high-throughtput organoid microinjector system has been developed that can deliver microbial communities into the organoid lumen (35).

The first tissue 3D organoid models used to study host–pathogen interactions were intestinal organoids (36). To date, lung organoids have been used in microbial infection studies to understand the molecular mechanisms of epithelial renewal upon viral infection (37) and to study the cytokine profile released in response to pattern recognition receptor activation by *Pseudomonas aeruginosa* (38). Here, wild-type and transgenic lung organoids were treated with bacterial flagellar hook proteins eliciting IL-1β and IL-6 release (38). Another study using organoids infected with *Cryptosporidium* oocytes provided deep understanding of the microbial life cycle and showed that the parasite is able to infect secretory and non-secretory cells, triggering the Type I interferon release from epithelial cells (39). Of note, even though organoids have reproduced the 3D lung architecture during infection, a role for immune cells has not yet been evaluated.

While the above-mentioned studies have been based on the use of 3D organoids differentiated from murine adult stem cells, several efforts are ongoing to generate organoids from immature lung epithelial and iPSCs. iPSCs are obtained by transfecting and reprogramming adult somatic cells with pluripotency transcription factors (40).

As well as studying host–pathogen interactions, lung organoids have proven valuable in understanding cystic fibrosis pathology (41, 42). Here, major breakthroughs have been achieved through using iPSCs derived from patients carrying genetic mutations to generate organoids. These organoids modeling cystic fibrosis have permitted drug testing directly on patient cells with affected organ properties (43). Further studies that aim to elucidate the molecular nature of the protective immune barriers in the lower and upper respiratory tracts will rapidly advance the rational design of novel therapeutics targeting such important diseases. Future studies using patient-specific organoids may permit bio-banking and the development of personalized medicines and targeted therapies for opportunistic pulmonary infections.

## Using Lung Organoids to Delineate IL-17R Signaling in Epithelial Lung Cells

Recent studies have identified the importance and complexity of interleukin-17 receptor (IL-17R) signaling by lung epithelial cells and highlighted the need for deeper investigations into the regulatory network activated by IL-17 cytokines in acute or chronic inflammation. Thus far, studies have shown that high IL-17R expression on lung epithelial cells has a prominent role in the innate immune defense against pulmonary fungal pathogens, including *Blastomyces dermatitidis* (44) and *Aspergillus fumigatus* (45). These epithelial cells may orchestrate innate antifungal immunity by first up-regulating the number of lymphocytes that secrete interleukin-17A (IL-17A) and granulocyte-macrophage colony-stimulating factor (GM-CSF) (44). They then respond to secreted IL-17 *via* the IL-17R, which regulates the secretion of antimicrobial peptides and chemokines that recruit neutrophils. IL-17R expressed on lung club cells orchestrates neutrophil recruitment and *Klebsiella pneumonia* resistance (46).

IL-17A and IL-17F homodimers both bind the IL-17R subunits IL-17RA and IL-17RC (47–49).

Also, the human IL-17A/F heterodimer may bind the complex IL-17R (50) since it may mimic the IL-17A as well as IL-17F behaving as a two-face cytokine. During *Aspergillus* fungal infection, the fungus increases IL-17F expression, which subsequently induces IL-33 and IL-17RC expression on lung epithelial cells, especially in the context of IL-17RA deficiency (45).

A polarized lung epithelium is required for IL-17R expression and innate immune functions, such as mucus production (51). Because organoid cultures recapitulate tissue polarity, they thus provide an exciting possibility of using lung organoids to comprehensively investigate IL-17R signaling in the lung. Improving our understanding of IL-17R signaling by lung epithelial cells is likely to offer new opportunities to develop and test therapeutics for inflammatory diseases and identify new molecular targets to improve resistance to infections. Such work is important given that human studies have demonstrated the importance of IL-17-driven immunity in LRTI infections, with mutations in IL-17RA or IL-17RC conferring increased susceptibility to RTIs (52, 53). In addition, the immune-free organoid microenvironment favors IL-17RC signaling studies, as epithelial cells express high levels of IL-17RC compared to immune cells; furthermore, the best characterized IL-17A-targeted cells are non-immune cells, including epithelial cells and mesenchymal cells of the lung (51).

Another important area of research in which lung organoids are anticipated to be of value is in deciphering IL-17F function in asthma. It is produced by multiple cell types including bronchial epithelial cells. IL-17F mediates asthma *via* IL-17R binding on bronchial epithelial cells, eosinophils, fibroblasts and airway smooth muscle cells (54). A certain extent of pulmonary IL-17F, however, is also released by immune cells, which are not differentiated in 3D-organoids. This limitation may be compensated by using exogenous IL-17F or by co-culturing organoids with IL-17F-producing cells. It would be interesting to study the independent role of epithelial IL-17F in modulating airway remodeling, asthma and steroid resistance in 3D cultures. In addition, transgenic lung organoids for IL-17R subunits expression may be useful to better disentangle IL-17F receptor signaling (Figure 1). The need to elucidate the function of the receptor subunits comes from the evidence that IL-17RA or IL-17RC mutations have been also described in human fungal infections (52, 53). Clearly, there is a need to recapitulate the 3D structure of lung organoids with the appropriate cell mixture in order to properly investigate pulmonary intercellular networks and immune receptor signaling pathways as IL-17R.

## Author Contributions

GP, AL, MA, IT, and SSJ critically read, analyzed, and discussed the literature and conceived the outline of the manuscript. JF and TZ wrote the manuscript. All the authors edited the manuscript and provided valuable discussions and criticisms.

### Conflict of Interest Statement

The authors declare that the research was conducted in the absence of any commercial or financial relationships that could be construed as a potential conflict of interest.
